# Effect of Wuzi Yanzong prescription on oligoasthenozoospermia rats based on UPLC-Q-TOF-MS metabolomics

**DOI:** 10.1080/13880209.2022.2101670

**Published:** 2022-08-09

**Authors:** Zhimin Chen, Baohua Dong, Yunxiu Jiang, Ying Peng, Wenbing Li, Lingying Yu, Yongxiang Gao, Changjiang Hu

**Affiliations:** a State Key Laboratory of Southwestern Chinese Medicine Resources, School of Pharmacy, Chengdu University of Traditional Chinese Medicine, Chengdu, China; b Institute of Qinghai Tibetan Plateau, Southwest Minzu University, Chengdu, China; c International Education College, Chengdu University of Traditional Chinese Medicine, Chengdu, China; d Sichuan Neo-Green Pharmaceutical Technology Development Co., Ltd, Chengdu, China

**Keywords:** Compound content, biomarkers, metabolic pathway, mechanism

## Abstract

**Context:**

Wuzi Yanzong Prescription (WYP) has long been used to treat male infertility, with convincing clinical evidence. However, its mechanism of action is not clear.

**Objective:**

WYP chemical components were quantified by HPLC, and the effect on oligoasthenozoospermia rats was explored based on UPLC-Q-TOF-MS metabonomics technology.

**Materials and methods:**

The solution was extracted by alcohol and water; the content of eight components in the extracting solution was determined by HPLC. Twenty-four Sprague-Dawley rats were randomly divided into control (CG), model (MG) and administration (PG) groups. Oligoasthenozoospermia was induced for 30 days in all, but the CG with daily oral doses of 20 mg/kg *Tripterysium* glycosides (TG). PG was given daily oral doses of WYP solution (1.08 g/kg), CG and MG received the same volume of distilled water, all administered for 30 days. After the last administration, the serum samples were collected and detected by UPLC-Q-TOF-MS.

**Results:**

An HPLC method for simultaneous determination of chlorogenic acid (0.081%), ellagic acid (0.021%), hyperoside (0.032%), verbascoside (0.011%), isoquercitrin (0.041%), astragalin (0.026%), kaempferol (0.009%) and schisandrin (0.014%) was established. Moreover, the 74 potential biomarkers and eight metabolic pathways related to oligoasthenozoospermia were identified by multivariate analysis and metabolite profiling. WYP can regulate 36 markers, mainly involving amino acid metabolism, lipid metabolism and nucleotide metabolism.

**Discussion and conclusions:**

This is a simple and accurate method for quality control of WYP. It further enriches the potential mechanism of WYP in the treatment of oligoasthenozoospermia. It can provide a theoretical basis for the rational application of WYP.

## Introduction

Infertility and subfertility affect a significant proportion of humanity. According to the World Health Organisation (WHO) definition, infertility is a disease of the male or female reproductive system defined by the failure to achieve a pregnancy after 12 months or more of regular unprotected sexual intercourse. Population-based studies have reported that about 15% of couples may suffer from infertility, thus representing a considerable issue for the global health community. In this context, approximately 40%– 50% of infertile couples are unable to conceive as a consequence of male reproductive impairment (Capogrosso et al. 2021; Pillai and McEleny 2021). In the male reproductive system, infertility is most commonly caused by problems in the ejection of semen, absence or low levels of sperm, or abnormal shape (morphology) and movement (motility) of the sperm. Oligoasthenospermia is caused by a variety of diseases or factors and usually manifests itself as ‘spermlessness’, ‘heirlessness’, ‘sterility’, ‘infertility’, ‘cold sperm’, ‘clear sperm’, ‘low sperm’, etc. (Fuxing et al. 2020). It is the most common type of semen abnormalities in male infertility patients (Wang et al. 2021).

At present, the treatment of male infertility by western medicine mainly includes the treatment of primary diseases, semen drugs, assisted reproductive technology, etc., but its therapeutic effects are limited and cannot fundamentally solve the problem of male infertility. The theory of traditional Chinese medicines (TCM) believes that male infertility with abnormal semen and few weak sperm is often caused by insufficient kidney essence, and should take the method of nourishing the kidney and improving the essence. A classical TCM formula for this type of infertility, Wuzi Yanzong prescription (WYP), was first recorded in the ‘she sheng zong miao fang’ text of the Ming Dynasty and is a mixture of *Lycii fructus* (the dried ripe fruit *Lycium barbarum* L.), stir-fried *cuscutae semen* (the dried ripe seed of *Cuscuta australis* R.Br. or *Cuscuta chinensis* Lam.), *rubi fructus* (the dried fruit of *Rubus chingii* Hu), saltwater stir-fried *plantaginis semen* (the dried ripe seed of *Plantago asiatica* L. or *Plantago depressa* Willd.) and steamed *schisandrae chinensis fructus* [the dried ripe fruit of *Schisandra chinensis* (Turcz.) Baill.]. It nourishes the kidney and strengthens the essence and therefore has been used to treat oligoasthenozoospermia secondary to kidney essence insufficiency as ‘The Number One Herbal Formula for Assisting Fertility’ (Yang et al. 2019). Modern studies have shown that WYP has a good protective effect on reproductive system (Sheng et al. 2018; Hu et al. 2019), immune system (Zhuo et al. 2019) and nervous system (Ruixue et al. 2019).

In the early stage, we have carried out research on WYP’s chemical composition, pharmacodynamics and other aspects. The results showed that WYP could restore testicular structure and significantly increase the serum levels of GnRH, LH, E2, T and decrease FSH, TGf-β1, Smad2 and Sma4 expression levels (Yang et al. 2019). The main components of WYP include polysaccharide, fatty acids, flavonoids, phenylpropanoids, organic acids, alkaloids, terpenoids, etc. But the research is relatively single and scattered, which cannot reflect the holistic view of WYP in the treatment of diseases. Therefore, this study intends to explore the intervention effect and regulatory mechanism of WYP on oligoasthenozoospermia rats based on ultra-high performance liquid chromatography-quadrupole time-of-flight mass spectrometry (UPLC-Q-TOF-MS) metabonomics to more comprehensively elaborate the metabolic pathway of WYP in the treatment of oligoasthenozoospermia, and provide reference for the clinical treatment of male reproductive system diseases.

## Materials and methods

### Plant materials

*Lycii fructus* (the dried ripe fruit *Lycium barbarum*, Lot no. 171101), *cuscutae semen* (the dried ripe seed of *Cuscuta australis*, Lot no. 180106), *schisandrae chinensis fructus* (the dried ripe fruit of *Schisandra chinensis*, Lot no. 170203), *rubi fructus* (the dried fruit of *Rubus chingii*, Lot no. 181023) and *plantaginis semen* (the dried ripe seed of *Plantago asiatica*, Lot no. 180405) were provided by Neo-Green Pharmaceutical Co., Ltd. (Chengdu, China) and were identified by Professor Changjiang Hu (Chengdu University of TCM, Chengdu, China).

### Chemicals and reagents

The reference standards of chlorogenic acid (Lot no. 110753-202018), kaempferol (Lot no. 110861-202013), hyperoside (Lot no. 110861-202013), verbascoside (Lot no. 111530-201914), schisandrin (Lot no. 110857-201815) and isoquercitrin (Lot no. 111809-201804) were obtained from the National Institutes for National Institute for Food and Drug Control (Beijing, China). Ellagic acid (Lot no. CHB-R-039), quercetin (Lot no. CHB-H-040) and astragalin (Lot no. CHB-Z-050) were obtained from the Chengdu Chroma-Biotechnology Co., Ltd. Tripterysium glycosides (TG) tablets (Lot no. 20171104) were purchased from Hunan Qianjin Xieli Pharmaceutical Co., Ltd. (Hunan, China).

#### Preparation of WYPextracting solution

Stir-fried *cuscutae semen*, saltwater stir-fried *plantaginis semen* and steamed *schisandrae chinensis fructus* were prepared according to Pharmacopoeia of the People's Republic of China 2020 Vision (ChP 2020). *Lycii fructus*, stir-fried *cuscutae semen*, *rubi fructus*, steamed *schisandrae chinensis fructus* and saltwater stir-fried *plantaginis semen* were combined in the proportion of 8:8:4:2:1 to form WYP (200 g), as outlined in the ChP 2020. WYP extracting solution was obtained by reflux exaction, adding six times 95%, 70% and 50% ethanol for 1.5 h each time. The dregs were refluxed successively adding six times water and four times water for 1.5 h each time. The filtrates were mixed five times. The decoction was concentrated to 463 mL by vacuum concentration.

### Animals

Twenty-four SD rats (male, 220 ± 20 g) were provided by Chengdu Dashuo Experimental Animal Co., Ltd. (Chengdu, China). All animals were housed under controlled temperature (25 ± 2 °C) and a 12-h light/dark cycle and fed standard diet and water. The animal experiments were reviewed by the Committee of Scientific Research and the Committee of Animal Care of the Chengdu University of TCM. All the animals were allowed to acclimatize in metabolism cages for 1 week prior to treatment.

### Establishment of oligoasthenozoospermia model and treatment

The rats were randomly divided into three groups, including control group (CG, *n* = 8), model group (MG, *n* = 8) and administration group (PG, *n* = 8). Oligoasthenozoospermia was induced for 30 days in all but the CG with daily oral doses of 20 mg/kg TG in the morning (Yang et al. 2019). Meanwhile, PG was given daily oral doses of 1.08 g/kg extracting solution of WYP, while the CG rats and model rats received the same volume of distilled water. On the 31st day, blood samples were collected from the abdominal aorta of all the rats after 45 min of intragastric administration. Prior to this, all of the rats were prohibited any food for 12 h before the last administration, but were allowed free access to water. The blood samples were left at room temperature for 1 h, and were centrifuged at 3500 rpm for 10 min at 4 °C. Then the supernatant was transferred into frozen pipes and stored at −80 °C until analysis.

### Component analysis of WYP extracting solution

#### Standard solution

A mixed stock solution containing reference standards was prepared by dissolving weighed samples of each compound in methanol, yielding chlorogenic acid 19.680 μg/mL, ellagic acid 20.928 μg/mL, hyperoside 23.040 μg/mL, isoquercitrin 19.200 μg/mL, verbascoside 21.984 μg/mL, astragalin 19.776 μg/mL, quercetin 25.248 μg/mL, kaempferol 19.584 μg/mL and schisandrin 20.736 μg/mL. Calibration curves were established by further dilution with methanol to six different concentrations measured by HPLC.

#### Sample preparation

Take about 1 mL of WYP extracting solution (to a 10 mL volumetric flask), adding a moderate amount of methanol, dilute to 10 mL. The material was filtered (0.45 μm pore size) before HPLC injection.

#### HPLC analysis

Chromatographic separation was performed to control the quality of the sample. HPLC analysis method refers to the conditions established by the research group (Yu-jiao et al. 2021). The main components in the sample were quantified on an UltiMate 3000 HPLC system (Thermo Fisher Scientific) using an Agilent 5 TC-C18 column (4.6 mm × 250 mm, 5 μm). The HPLC separation conditions were as follows: flow velocity 1.0 mL/min, injected volume 5 μL, column temperature 40 °C and UV wave length 254 nm. The mobile phases A (acetonitrile) and B (0.04% phosphoric acid aqueous) (V/V). The gradient elution was as follows: 0–5 min, 5%–15%A; 5–10 min, 15%–17%A; 10 ∼ 25 min, 17%A; 25–35 min, 17%–26%A; 35–60 min, 26%–56%A.

### Metabolomic study

#### Metabolite extraction

A blood sample (100 μL) was transferred to an Eppendorf (EP) tube, and 400 μL extract solution (acetonitrile: methanol = 1: 1) (V/V) containing isotopically-labelled internal standard mixture was added. After 30-s vortex, the samples were sonicated for 5 min in ice-water bath. Then the samples were incubated at −40 °C for 1 h and centrifuged at 12,000 rpm for 15 min at 4 °C. Supernatant (400 μL) was transferred to a fresh tube and dried in a vacuum concentrator at 37 °C. Then, the dried samples were reconstituted in 200 μL of 50% acetonitrile by sonication on ice for 10 min. The constitution was then centrifuged at 13,000 rpm for 15 min at 4 °C, and 75 μL of supernatant was transferred to a fresh glass vial for LC/MS analysis. The quality control (QC) sample was prepared by mixing an equal aliquot of the supernatants from all of the samples.

#### Metabolite analysis

The UPLC separation was carried out using a 1290 Infinity series UPLC System (Agilent Technologies), equipped with a UPLC BEH Amide column (2.1 × 100 mm, 1.7 μm, Waters). The mobile phase consisted of 25 mmol/L ammonium acetate and 25 mmol/L ammonia hydroxide in water (pH = 9.75) (A) and acetonitrile (B). The analysis was carried with elution gradient as follows: 0–0.5 min, 95%B; 0.5–7.0 min, 95%–65% B; 7.0–8.0 min, 65%–40% B; 8.0–9.0 min, 40% B; 9.0–9.1 min, 40%–95% B; 9.1–12.0 min, 95% B. The flow velocity was 1.0 mL/min. The column temperature was 25 °C. The auto-sampler temperature was 4 °C, and the injection volume was 2 μL (pos) or 2 μL (neg), respectively.

The Triple TOF 6600 mass spectrometry (AB Sciex) was used for its ability to acquire MS/MS spectra on an information-dependent basis (IDA) during an LC/MS experiment. In this mode, the acquisition software (Analyst TF 1.7, AB Sciex) continuously evaluates the full scan survey MS data as it collects and triggers the acquisition of MS/MS spectra depending on preselected criteria. In each cycle, the most intensive 12 precursor ions with intensity above 100 were chosen for MS/MS at collision energy (CE) of 30 eV. The cycle time was 0.56 s. ESI source conditions were set as following: Gas 1 as 60 psi, Gas 2 as 60 psi, Curtain Gas as 35 psi, Source Temperature as 600 °C, Declustering potential as 60 V, Ion Spray Voltage Floating (ISVF) as 5000 V or −4000 V in positive or negative modes, respectively.

#### Data preprocessing and analysis

MS raw data (.wiff) files were converted to the mzXML format by ProteoWizard and processed by R package XCMS (version 3.2). The process includes peak deconvolution, alignment and integration. Minfrac and cut off are set as 0.5 and 0.3, respectively. The resultant data matrices were imported to SIMCA-P (V15.0.2) for the principal component analysis (PCA) and orthogonal partial least squares discriminant analysis (OPLS-DA). The PCA was used to evaluate the MS data in a first overview of the cluster of groups. The following analysis with OPLS-DA scores was performed to obtain the VIP scores and dataset of the relative intensity of metabolites to identify biomarkers. In-house MS2 database (Biotree biomedical technology co., LTD) was applied for metabolite identification. The combination approaches of Human Metabolome Database (http://www.hmdb.ca) and mzcloud (https://www.mzcloud.org) were applied to analyse biomarkers and relate biological information. The pathway analysis and inner relation among them were undertaken with MetaboAnalyst 5.0 (https://www.metaboanalyst.ca/) and KEGG database (http://www.kegg.jp/). The differences in metabolites among groups were compared by Student’s *t-*test, and *p* < 0.05 was considered statistically significant.

## Results

### Identification and determination of components from WYP extracting solution

**Figure 1.**
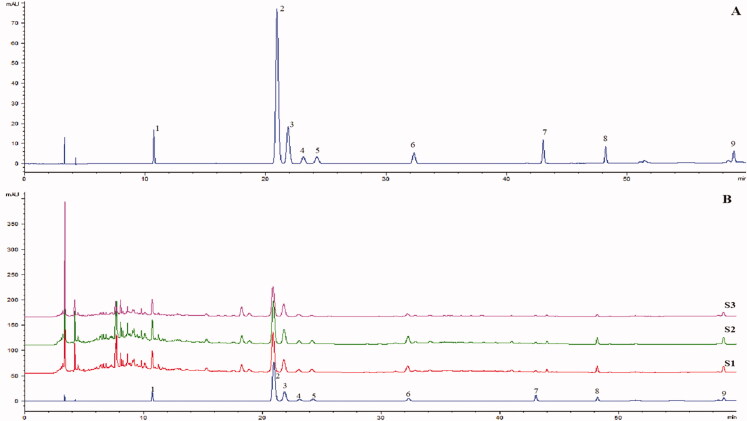
Characteristic chromatogram of reference substances (a) and WYP extracting solution (b). 1-Chlorogenic acid, 2-Ellagic acid, 3-Hyperoside, 4-Verbascoside, 5-Isoquercitrin, 6-Astragalin, 7-Quercetin, 8-Kaempferol, 9-Schisandrin.

The chromatograms of mixed standards and samples are shown in Figure 1. The chromatographic peaks are well separated, the retention time is accurate and the determination results are reliable. By comparing the retention time in HPLC analysis with an accepted standard, nine components were identified, including chlorogenic acid, kaempferol, hyperoside, verbascoside, schisandrin, isoquercitrin, ellagic acid, quercetin and astragalin. The contents of 8 components were determined. The results are shown in Table 1.

**Table 1.** The content of eight components in WYP extracting solution (%, *n* = 3).

Component	Chlorogenic acid	Ellagic acid	Hyperoside	Verbascoside	Isoquercitrin	Astragalin	Kaempferol	Schisandrin
Content	0.081	0.021	0.032	0.011	0.041	0.026	0.009	0.014

### Results of the serum metabolomics

#### Total ion flow chromatogram of rat serum

**Figure 2.**
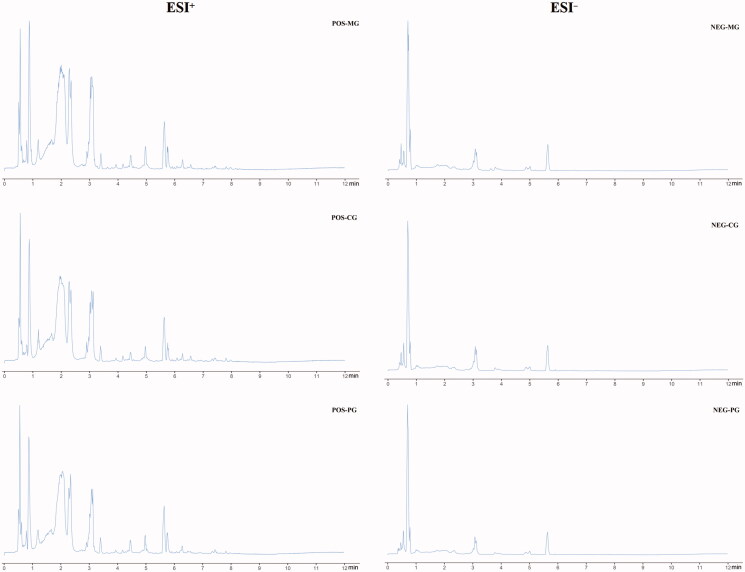
Total ion chromatograms of mouse serum in each group under different ion modes by UPLC-Q-TOF-MS.

UPLC-Q-TOF-MS was used to analyse the serum of CG, MG and PG. The total ion flow chromatogram is shown in Figure 2. The endogenous markers obtained excellent separation within 12 min. Further analysis of the total ion flow chromatograms under the positive and negative ion modes showed that the total ion flow chromatograms of the samples in each group were basically similar, but there were some differences in the peak type and peak area of each group, indicating that some metabolites in rats were changed. The reason for this difference may be that the endogenous metabolites and their metabolic pathways changed in rats before and after WYP treatment.

#### Multivariate analysis and metabolite profiling

**Figure 3.**
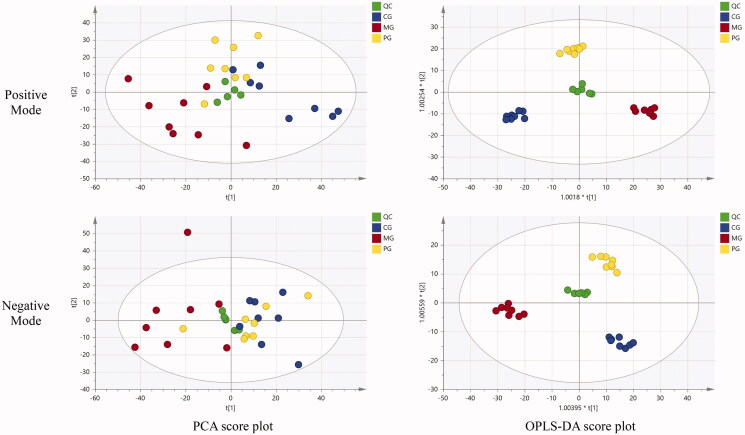
The results of multivariate data analysis. *n* = 8 rats/group.

As shown in Figure 3, the PCA score plot was introduced to distinguish and evaluate the status of each group. In the positive and negative mode, the distribution of QC samples is concentrated, showing good reproducibility and reliability. The data of MG and other groups were well distinguished, and the distance between the CG and MG was far, indicating that the model was successfully established. The CG had some discrete data, which may be caused by individual differences and injection time differences of rats. OPLS-DA is a noise reduction method based on partial least squares method, which can remove some variations in the independent variables that were not related to the dependent variables, reduce the complexity of model, enhance the model forecast capability and better reveal the differences between groups (Zhou et al. 2016). The OPLS-DA model was used to further differentiate the differences among the groups. As shown in Figure 3, The OPLS-DA score plots (R2X (cum) = 0.469, R2Y (cum) = 0.970, Q2 (cum) = 0.592 in positive mode and R2X (cum) = 0.357, R2Y (cum) = 0.946, Q2 (cum) = 0.535 in negative mode) showed the prediction ability of the model is good, and the metabolites in each group were completely separated either on the positive or negative mode. The samples in the PG were more inclined to the CG, indicating that WYP had a callback effect on the abnormal endogenous metabolites.

**Figure 4.**
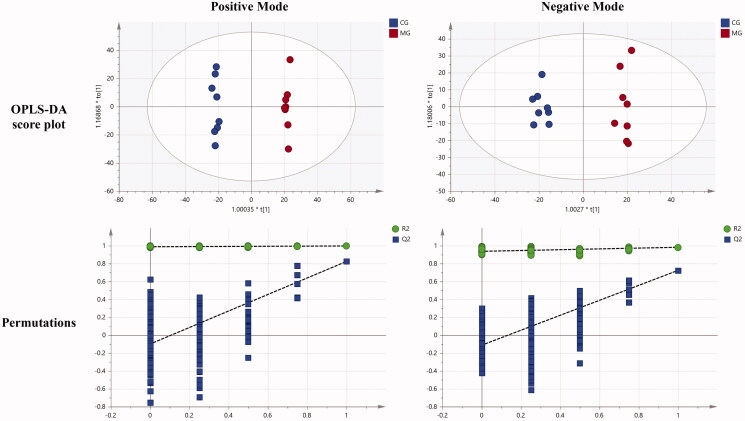
Scatter plots of OPLS-DA and permutation tests of OPLS-DA models for CG vs. MG.

To gain a better discrimination between the CG and MG, the OPLS-DA approaches were applied to differentiate the metabolic profile and find the potential biomarkers. The OPLS-DA score plots (R2X (cum) = 0.424, R2Y (cum) = 0.997, Q2(cum) = 0.824 in positive mode and R2X (cum) = 0.285, R2Y (cum) = 0.982, Q2(cum) = 0.724 in negative mode) showed an overall goodness of fit between the MG and CG (Figure 4). The results of 200 permutation tests showed that the intercept of Q2 regression line is less than 0, which indicates that the model has not been fitted, and the OPLS-DA model is reliable and stable. The metabolites were identified under the reference standard of same retention times and same molecular weights, while *p*-values less than 0.05. Based on the protocols described above and the comparison of the metabolites between the CG and MG, 74 differential biomarkers were identified, which could be considered as the endogenous biomarkers of oligoasthenozoospermia. Forty-four endogenous metabolites were regulated significantly by WYP (*p* < 0.05). The *p*-values were derived using a one-way ANOVA. The results are shown in Table 2.

**Table 2.** The effect of WYP on potential biomarkers in serum of rats with oligoasthenozoospermia.

Mode	NO.	Name	MS2 score	m/z	Retention times	MS/MS	HMDB	KEGG	VIP	MG vs CG	PG vs MG
**Positive Mode**	1	L-Alanine	0.9486	90.0549	373.134	90,44	HMDB0000161	C00041	1.75332	↓**	↑*
	2	Cytosine	0.9787	112.0508	204.459	112,95,69	HMDB0000630	C00380	1.62441	↓**	—
	3	epsilon-Caprolactam	0.9201	114.0911	47.224	114,96	HMDB0062769	C06593	1.16769	↓*	—
	4	Betaine	0.9928	118.0859	352.114	118,101,59	HMDB0000043	C00719	1.27632	↑**	↓*
	5	L-Pipecolic acid	0.9861	130.0864	497.814	130.84	HMDB0000716	C00408	1.74143	↓**	↑**
	6	Ornithine	0.9982	133.0968	487.025	133,116,70	HMDB0000214	C00077	1.81672	↓**	↑**
	7	L-Glutamine	0.9966	147.0762	394.632	147,130,101,84	HMDB0000641	C00064	1.32863	↓*	—
	8	L-Lysine	0.9949	147.1131	497.918	147,130,84	HMDB0000182	C00047	1.65945	↓**	↑**
	9	Methoxyacetic acid	0.9969	151.0611	152.169	73,45	HMDB0041929	NA	1.59632	↓**	**—**
	10	DL-Methionine sulfoxide	0.9968	166.0530	362.144	166,102,74,56	HMDB0002005	C02989	1.40454	↓**	↑**
	11	D-Pipecolinic acid	0.9946	171.1126	259.102	130,84	HMDB0005960	NA	1.20157	↓*	—
	12	L-Isoleucine	0.9194	173.1283	238.064	132,86	HMDB0000172	C00407	1.17020	↓*	—
	13	L-Tyrosine	0.9712	182.0802	331.276	182,165,136,123	HMDB0000158	C00082	1.21441	↓**	—
	14	4-Pyridoxic acid	0.9910	184.0605	44.731	184,166,148	HMDB0000017	C00847	1.27873	↑**	↓**
	15	NG,NG-dimethyl-L-arginine (ADMA)	0.9891	203.1507	469.610	203,158,116,88	NA	NA	1.53663	↓**	↑*
	16	DL-Phenylalanine	0.9839	207.1127	225.183	166,120,103	HMDB0000159	C00079	1.44641	↓**	**—**
	17	Pro-Val	0.9693	215.1389	290.156	215,169	HMDB0029030	NA	1.35812	↑**	↓*
	18	N6-Methyl-L-lysine	0.9167	221.1496	478.395	161,130	HMDB0002038	C02728	1.72671	↓**	↑**
	19	His-Ala	0.9543	227.1139	411.956	227,181,110	HMDB0028878	NA	1.47186	↓**	—
	20	Gemcitabine	0.9958	228.0635	164.067	264,163,112	HMDB0014584	C07650	1.18685	↓*	—
	21	Deoxycytidine	0.9985	228.0985	204.459	228,210,112	HMDB0000014	C00881	1.64840	↓**	—
	22	L-Anserine	0.9913	241.1297	387.914	241,170,109	HMDB0000194	C01262	1.41110	↓**	—
	23	Pro-Ser	0.9481	244.1293	370.512	203,98,70	HMDB0029026	NA	1.33933	↑*	↓*
	24	Glycerophosphocholine	0.9893	258.1113	377.232	258,184	HMDB0000086	C00670	1.33571	↑**	↓**
	25	Pro-Phe	0.9855	263.1393	236.452	263,217,166	HMDB0011179	NA	1.22300	↑*	↓*
	26	Thiamine	0.9644	265.1111	374.743	266,257	HMDB0000235	C00378	1.55975	↓**	↑*
	27	Ile-Arg	0.9497	288.2035	331.890	288,271,175	HMDB0028901	NA	1.40149	↓*	—
	28	L-Palmitoylcarnitine	0.9897	400.3438	164.937	400,341,85	HMDB0000222	C02990	1.57621	↑**	↓**
	29	Stearoylcarnitine	0.9773	428.3739	161.055	428,369	HMDB0000848	NA	1.75572	↑**	↓**
	30	Glycodeoxycholic acid	0.9183	450.3212	204.252	450,432,414	HMDB0000631	C05464	1.12344	↑*	—
	31	1-Palmitoyl-2-hydroxy-*sn*-glycero-3-phosphoethanolamine	0.9828	454.2939	193.894	454,436,313	HMDB0011503	NA	1.54976	↑**	↓*
	32	1-Myristoyl-*sn*-glycero-3-phosphocholine	0.9867	468.3092	191.364	468,450,184	HMDB0010379	NA	1.68555	↑**	↓*
	33	1-Palmitoyl-*sn*-glycero-3-phosphocholine	0.9892	496.3419	186.941	496,478,184	HMDB0010382	C04230	1.73299	↑**	↓**
	34	Taurodeoxycholic acid	0.9502	517.3299	141.905	500,482,464	HMDB0000896	C05463	1.22359	↓*	—
	35	1-Oleoyl-*sn*-glycero-3-phosphocholine	0.9793	522.3555	184.142	522,504,184	HMDB0002815	C04230	1.59291	↑**	↓*
	36	Ile-Asn	0.9790	529.2428	469.300	246,229,133	HMDB0028902	NA	1.57365	↓**	—
	37	1-*O*-(*cis*-9-Octadecenyl)-2-*O*-acetyl-*sn*-glycero-3-phosphocholine	0.9595	592.4342	177.233	549	NA	NA	1.51588	↑**	↓*
	38	PC(16:0/16:0)	0.9979	756.5548	125.348	734,184	HMDB0000564	C00157	1.65764	↑**	↓**
	39	Thioetheramide-PC	0.9602	780.5542	142.467	788	NA	NA	1.54014	↑**	↓*
	40	Sphingomyelin (d18:1/18:0)	0.9996	794.6068	138.075	732,184	HMDB0001348	C00550	1.19329	↑*	—
**Negative Mode**	41	Thymine	0.9991	125.0338	103.323	125,89,75	HMDB0000262	C00178	2.06874	↓**	↑*
	42	Cyanuric acid	0.9997	129.0167	238.152	128,85,42	HMDB0041861	C06554	1.14547	↑*	—
	43	Glycerol 3-phosphate	0.9992	171.0097	41.069	171,114	HMDB0000126	C00093	1.85724	↑**	↓*
	44	Salicylic acid	0.9919	174.9785	24.956	137,93	HMDB0001895	C00805	1.18343	↑*	↓*
	45	Allantoic acid	0.9050	175.0445	353.533	175,131,115,89	HMDB0001209	C00499	1.35403	↓*	↑**
	46	Hippuric acid	0.9752	178.0482	190.021	178,160,134,77	HMDB0000714	C01586	1.43505	↑**	↓*
	47	Indoleacrylic acid	0.9935	186.0532	106.929	186,142,116	HMDB0000734	NA	1.49639	↑*	—
	48	Dihydrothymine	0.9432	187.0701	394.733	127,41	HMDB0000079	C00906	1.56351	↓**	—
	49	3-Indolepropionic acid	0.9472	188.0692	104.418	188,173,144	HMDB0002302	NA	1.34632	↑*	—
	50	*sn*-Glycerol 3-phosphoethanolamine	0.9388	214.0457	386.448	214,153.79	HMDB0059660	NA	1.63793	↑**	↓*
	51	2′-Deoxyuridine	0.9675	227.0643	116.430	227,184,137	HMDB0000012	C00526	1.37177	↓*	—
	52	Thymidine	0.9951	241.0803	101.818	241,151,125	HMDB0000273	C00214	1.95515	↓**	↑*
	53	Pentadecanoic Acid	0.9876	241.2140	44.329	241,223	HMDB0000826	C16537	1.65875	↑**	↓*
	54	Phosphorylcholine	0.9825	242.0764	376.906	182,79	HMDB0001565	C00588	1.92395	↑**	↓**
	55	gamma-L-Glutamyl-L-glutamic acid	0.9629	275.0851	459.653	275,257,146,128,101	HMDB0011737	C05282	1.58273	↑**	↓*
	56	Xanthosine	0.9962	283.0644	304.953	283,151,108	HMDB0000299	C01762	1.52693	↑**	↓*
	57	Pristanic acid	0.9888	297.2760	41.143	297,279,253,225	HMDB0000795	NA	1.35463	↑*	—
	58	Eicosapentaenoic Acid	0.9379	301.2138	42.379	301,257,203	HMDB0001999	C06428	1.25931	↑*	—
	59	11(*Z*),14(*Z*)-Eicosadienoic Acid	0.9922	307.2604	41.046	307,289	NA	NA	1.89887	↑**	**—**
	60	2*E*-Eicosenoic acid	0.9993	309.2768	40.552	309,291	NA	NA	1.44048	↑**	**—**
	61	Docosahexaenoic acid	0.9777	327.2302	41.799	327,283,229	HMDB0002183	C06429	1.45903	↑*	**—**
	62	Docosatrienoic Acid	0.9231	333.2741	39.732	333,315,289	HMDB0002823	NA	1.22830	↑*	—
	63	Erucic acid	0.9990	337.3080	39.793	337,319	HMDB0002068	C08316	1.64180	↑**	↓**
	64	Behenic acid	0.9517	339.3222	39.656	339	HMDB0000944	C08281	1.28897	↑*	↓*
	65	Nervonic acid	0.9999	365.3392	39.668	365,347	HMDB0002368	C08323	1.45718	↑**	↓**
	66	Thromboxane B2	0.9777	369.2233	168.145	369,289,195,177,169,159	HMDB0003252	C05963	1.71803	↑**	↓**
	67	Lithocholic acid	0.9907	375.2849	46.817	375	HMDB0000761	C03990	1.31947	↑*	—
	68	1-Palmitoyl Lysophosphatidic Acid	0.9755	409.2304	243.598	409,391	NA	NA	1.50878	↑*	—
	69	1-Palmitoyl-2-hydroxy-*sn*-glycero-3-phosphoethanolamine	0.9811	452.2732	192.883	452,434,391	NA	NA	1.61505	↑**	↓*
	70	1-Oleoyl-L-.alpha.-lysophosphatidic acid	0.9738	457.2302	237.504	170,151	NA	NA	1.32805	↑*	↓*
	71	11-Keto-.beta.-boswellic acid	0.9192	469.3267	47.874	407,391,451	NA	NA	2.02895	↑**	↓**
	72	Maslinic Acid	0.9999	471.3417	47.409	471,407	HMDB0002392	C16939	1.76930	↑**	↓**
	73	Arachidonic Acid	0.9794	607.4669	41.181	303,304,259	HMDB0001043	C00219	1.76391	↑**	↓**
	74	20-HETE	0.9094	639.4567	47.186	301,273,245	HMDB0005998	C14748	1.78454	↑**	↓**

*p < 0.05; **p < 0.01.

#### Metabolic pathway analysis

**Figure 5.**
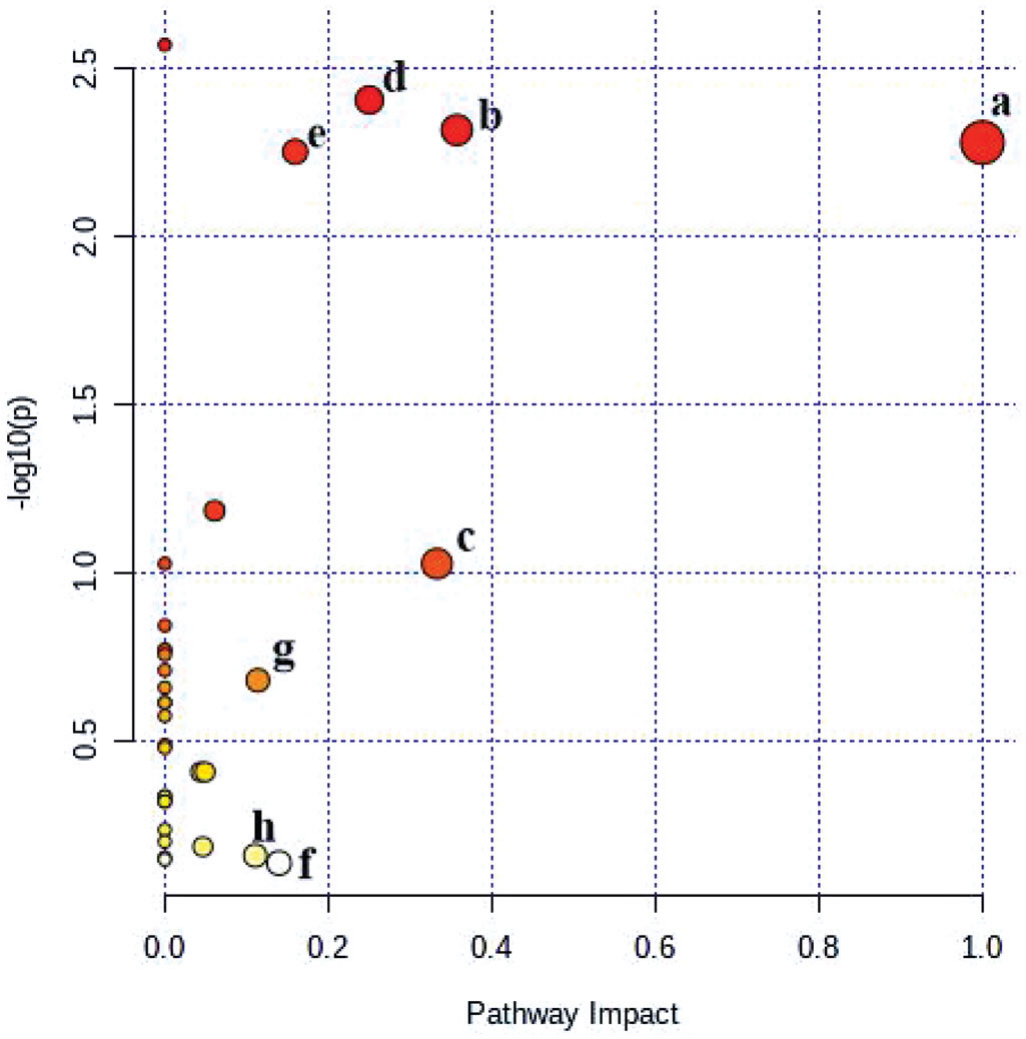
The metabolic pathway analysis of the SYD samples. (a-Phenylalanine, tyrosine and tryptophan biosynthesis, b-Phenylalanine metabolism, c-Arachidonic acid metabolism, d-Glycerophospholipid metabolism, e-Pyrimidine metabolism, f-Tyrosine metabolism, g-Alanine, aspartate and glutamate metabolism, h-Arginine and proline metabolism).

To explore the potential metabolic pathways of oligoasthenozoospermia, 68 endogenous biomarkers were imported into Metaboanalyst 5.0. The pathway impact greater than 0.1 was used as the standard to screen the main metabolic pathway. As shown in Table 3 and Figure 5, 8, corresponding pathways, which could be positively related to oligoasthenozoospermia were constructed, including the phenylalanine, tyrosine and tryptophan biosynthesis, phenylalanine metabolism, arachidonic acid metabolism, glycerophospholipid metabolism, pyrimidine metabolism, and tyrosine metabolism.

**Table 3.** The enrichment analysis of encyclopedia of Genes and Genomes (KEGG) metabolic pathways.

No.	Pathway	Total	Hits	Impact	Pathway ID	Class
1	Phenylalanine, tyrosine and tryptophan biosynthesis	4	2	1.000	00400	Metabolism; Amino acid metabolism
2	Phenylalanine metabolism	12	3	0.357	00360	Metabolism; Amino acid metabolism
3	Arachidonic acid metabolism	36	3	0.333	00590	Metabolism; Lipid metabolism
4	Glycerophospholipid metabolism	36	5	0.250	00564	Metabolism; Lipid metabolism
5	Pyrimidine metabolism	39	5	0.159	00240	Metabolism; Nucleotide metabolism
6	Tyrosine metabolism	42	1	0.140	00350	Metabolism; Amino acid metabolism
7	Alanine, aspartate and glutamate metabolism	28	2	0.114	00250	Metabolism; Amino acid metabolism
8	Arginine and proline metabolism	38	1	0.111	00330	Metabolism; Amino acid metabolism

## Discussion

As a unique theory of TCM, syndrome differentiation treatment is the soul of the basic theory of TCM and plays an important role in the diagnosis of TCM. To study the mechanism of WYP, we must first establish relevant animal pathological models. The previous research showed that the establishment of oligoasthenozoospermia model by intragastric administration of TG had high success rate, simple operation and good repeatability (Huangfu et al. 2014; Yang et al. 2019). To ensure the stability of the main chemical components in WYP extracts solution, we established an analytical method to identify the eight components, the results showed that the eight components showed good linearity in their respective ranges. Furthermore, through the analysis of serum metabolomics, 74 potential biomarkers and eight metabolic pathways were identified to be related to oligoasthenozoospermia. It is mainly related to amino acid metabolism, lipid metabolism and nucleotide metabolism. This is consistent with the literature report that WYP can regulate the concentration of amino acids, lipids and other metabolites of non-obstructive oligoasthenospermia, normalize the metabolic phenotype and regulate metabolic disorders (Zou et al. 2019).There are many TCM syndrome types. The model of oligoasthenospermia prepared by TG in this paper belongs to the syndrome of deficiency of kidney essence. Compared with the model of orchiectomy, it has a wider scope of application and more representative significance, which enriches the research on the mechanism of WYP.

According to the enriched metabolic pathway, the most significant metabolic feature of oligoasthenozoospermia is the change of amino acid metabolism. A variety of amino acid metabolic pathways, including phenylalanine, tyrosine and tryptophan biosynthesis, phenylalanine metabolism, tyrosine metabolism, alanine, aspartate and glutamate metabolism, arginine and proline metabolism, suggest that amino acids play an important role in spermatogenesis, development and maturation. Free amino acids participate in the cellular metabolism of motile sperm and are the source of energy supplement, which can promote its activity and reduce the harmful effects of toxins. Essential amino acids are important raw materials for the synthesis of nucleic acids. Supplementing essential amino acids will improve the body's ability to synthesize nucleic acids and promote spermatogenesis (Han et al. 2017).

In addition to amino acid metabolism, lipid metabolism and nucleoside metabolism also play a very important role in oligoasthenozoospermia, such as arachidonic acid metabolism, glycerophospholipid metabolism and pyrimidine metabolism. Modern research shows that lipid is an important part of sperm membrane, which participates in the changes of sperm structure in the process of spermatogenesis, and lipid metabolism provides energy for sperm (Hua et al. 2020). As the important components of various biofilms, fatty acids and cholesterol deletion will cause sperm damage (Huiqing et al. 2017). Other studies have shown that abnormal arachidonic acid metabolism can reduce sperm motility by activating p38 MAPK pathway through lipoxygenase, cytochrome P450 and cyclooxygenase pathways (Yu et al. 2019). This study found that WYP can improve metabolic disorder by regulating 36 biomarkers. It is suggested that WYP activity depends on multiple chemical components and effectively acts on multiple targets reflecting different potential pathways. In future research, the association of components, targets and efficacy can be realized through the research on serum pharmacochemistry and network pharmacology of WYP, combined with data analysis technologies to provide more clear and sufficient evidence for the mechanism of WYP, and to provide a basis for clinical application of WYP.

## Conclusions

In this study, a method for the determination of eight components in WYP extracting solution was established. The method is simple, stable and feasible, and can be used for the quality control of WYP. WYP mainly treats oligoasthenozoospermia by regulating amino acid metabolism, lipid metabolism and nucleotide metabolism. It further enriches the potential mechanism of WYP in the treatment of oligoasthenozoospermia. It can provide a theoretical basis for the rational application of WYP in clinic.
